# Sodium-activated potassium channels shape peripheral auditory function and activity of the primary auditory neurons in mice

**DOI:** 10.1038/s41598-019-39119-z

**Published:** 2019-02-22

**Authors:** Daniël O. J. Reijntjes, Jeong Han Lee, Seojin Park, Nick M. A. Schubert, Marcel van Tuinen, Sarath Vijayakumar, Timothy A. Jones, Sherri M. Jones, Michael Anne Gratton, Xiao-Ming Xia, Ebenezer N. Yamoah, Sonja J. Pyott

**Affiliations:** 1 University of Groningen, University Medical Center Groningen, Groningen, Department of Otorhinolaryngology and Head/Neck Surgery, 9713GZ Groningen, The Netherlands; 2 Department of Physiology and Cell Biology, Program in Communication and Sensory Sciences, School of Medicine, University of Nevada Reno, Reno, NV 89557 USA; 3 University of Nebraska Lincoln, Department of Special Education and Communication Disorders, 304B Barkley Memorial Center, Lincoln, NE 68583 USA; 4 Departments of Otorhinolaryngology Head and Neck Surgery and Anesthesiology, Washington University School of Medicine, St. Louis, MO 63110 USA

## Abstract

Potassium (K^+^) channels shape the response properties of neurons. Although enormous progress has been made to characterize K^+^ channels in the primary auditory neurons, the molecular identities of many of these channels and their contributions to hearing *in vivo* remain unknown. Using a combination of RNA sequencing and single molecule fluorescent *in situ* hybridization, we localized expression of transcripts encoding the sodium-activated potassium channels K_Na_1.1 (SLO2.2/Slack) and K_Na_1.2 (SLO2.1/Slick) to the primary auditory neurons (spiral ganglion neurons, SGNs). To examine the contribution of these channels to function of the SGNs *in vivo*, we measured auditory brainstem responses in K_Na_1.1/1.2 double knockout (DKO) mice. Although auditory brainstem response (wave I) thresholds were not altered, the amplitudes of suprathreshold responses were reduced in DKO mice. This reduction in amplitude occurred despite normal numbers and molecular architecture of the SGNs and their synapses with the inner hair cells. Patch clamp electrophysiology of SGNs isolated from DKO mice displayed altered membrane properties, including reduced action potential thresholds and amplitudes. These findings show that K_Na_1 channel activity is essential for normal cochlear function and suggest that early forms of hearing loss may result from physiological changes in the activity of the primary auditory neurons.

## Introduction

Encoding of auditory signals in the cochlea by the primary auditory neurons, the spiral ganglion neurons (SGNs), requires a repertoire of ion channels to establish the variation in response properties that are essential for normal hearing. Potassium (K^+^) channels are especially important in determining both active and passive membrane properties, including resting membrane potentials as well as action potential thresholds, durations, firing rates and timing. Thus, K+ channels are critical determinants of the response properties of the SGNs. Although enormous progress has been made to characterize K^+^ channels in SGNs^1–4^, the molecular identities of many of these channels and their contributions to hearing *in vivo* remain unknown.

To accelerate the discovery of K^+^ channels that regulate encoding of auditory signals as part of the afferent signalling complex^3^, we used RNA sequencing to obtain transcriptomes from the intact sensorineural structures, including the organ of Corti and SGNs, isolated from adult mice. In prioritizing identified K^+^ channels for further functional investigation, we were especially interested in the subset that belongs to the SLO family of K^+^ channels. These channels are distinguished by their relatively large single channel conductance, regulation by intracellular ions, and/or activation by membrane potential^5^. The dual regulation of these channels by intracellular ions and membrane potential positions these channels at the interface of signalling pathways, and, not surprisingly, members of this family are known to regulate a variety of functions. These channels include K_Ca_1.1 (SLO1/BK), K_Na_1.1 (SLO2.2/Slack), K_Na_1.2 (SLO2.1/Slick) and K_Ca_5.1 (SLO3).

Examination of the contribution of SLO K^+^ channels to the peripheral auditory system has been limited to K_Ca_1.1, which is regulated by intracellular Ca^2+^ and membrane voltage. The K_Ca_1.1 channel is abundantly expressed in inner and outer hair cells^6^ and likely also expressed in SGNs^7^. Mice lacking K_Ca_1.1 show subtle deficits in auditory function^8^ and specifically auditory encoding^7^. The role of the remaining family members, K_Ca_5.1, K_Na_1.1 and K_Na_1.2 is unknown. K_Ca_5.1, which is regulated by intracellular H^+^, is found in spermatocytes and necessary for male fertility^9–11^. K_Na_1.1 and K_Na_1.2, which are regulated by intracellular Na^+^ and Cl^−^, are found in a variety of neurons, especially those with action potentials triggered by Na^+^-influx^12^.

K_Na_1.1 and K_Na_1.2 have been examined in the central auditory system, where they are abundantly expressed in neurons of the medial nucleus of the trapezoid body (MNTB) in the auditory brainstem^13,14^. K_Na_1.1 and K_Na_1.2 are regulated by intracellular Na^+^ and, in neurons of the MNTB, manipulation of intracellular Na^+^ concentration and application of pharmacological activators indicate that K_Na_ activity improves the fidelity of timing at high action potential frequencies^15^. Outside of the central nervous system, K_Na_1.1 and/or 1.2 are expressed in the primary sensory neurons of the dorsal root ganglion neurons^16–20^. Genetic deletion of either K_Na_1.1^19^ or K_Na_1.2^20^ results in increased excitability of distinct populations of dorsal root ganglion (DRG) neurons and exacerbated nociceptor responses. These findings, expression of K_Na_ channels in primary sensory neurons and contribution of K_Na_ activity to signal encoding in the central auditory system, motivate examination of their role in regulating the function of the peripheral auditory system.

In this study, we investigated the expression of K_Na_1.1, K_Na_1.2, and K_Ca_5.1 in the inner ear. We localized K_Na_1 transcript expression to the sensorineural structures of the inner ear and specifically SGNs. We did not find evidence for expression of K_Ca_5.1 in the SGNs. We took advantage of K_Na_1.1/1.2 double knockout (DKO) mice to identify the contribution of K_Na_1 channels to function of the SGNs *in vivo* and determine the response properties of isolated SGNs *in vitro*. These findings indicate that K_Na_1 channels are essential for normal auditory function, by shaping activity of the primary auditory neurons. The data also suggest that early forms of hearing loss may result from physiological changes in the activity of the primary auditory neurons. This work highlights the utility of this experimental approach to inventory the ion channels that regulate encoding of auditory signals and identify their contributions to hearing.

## Results

### SLO channel transcripts encoding K_Na_1 channels are expressed in the intact sensorineural structures and specifically spiral ganglion neurons

**Figure 1:**
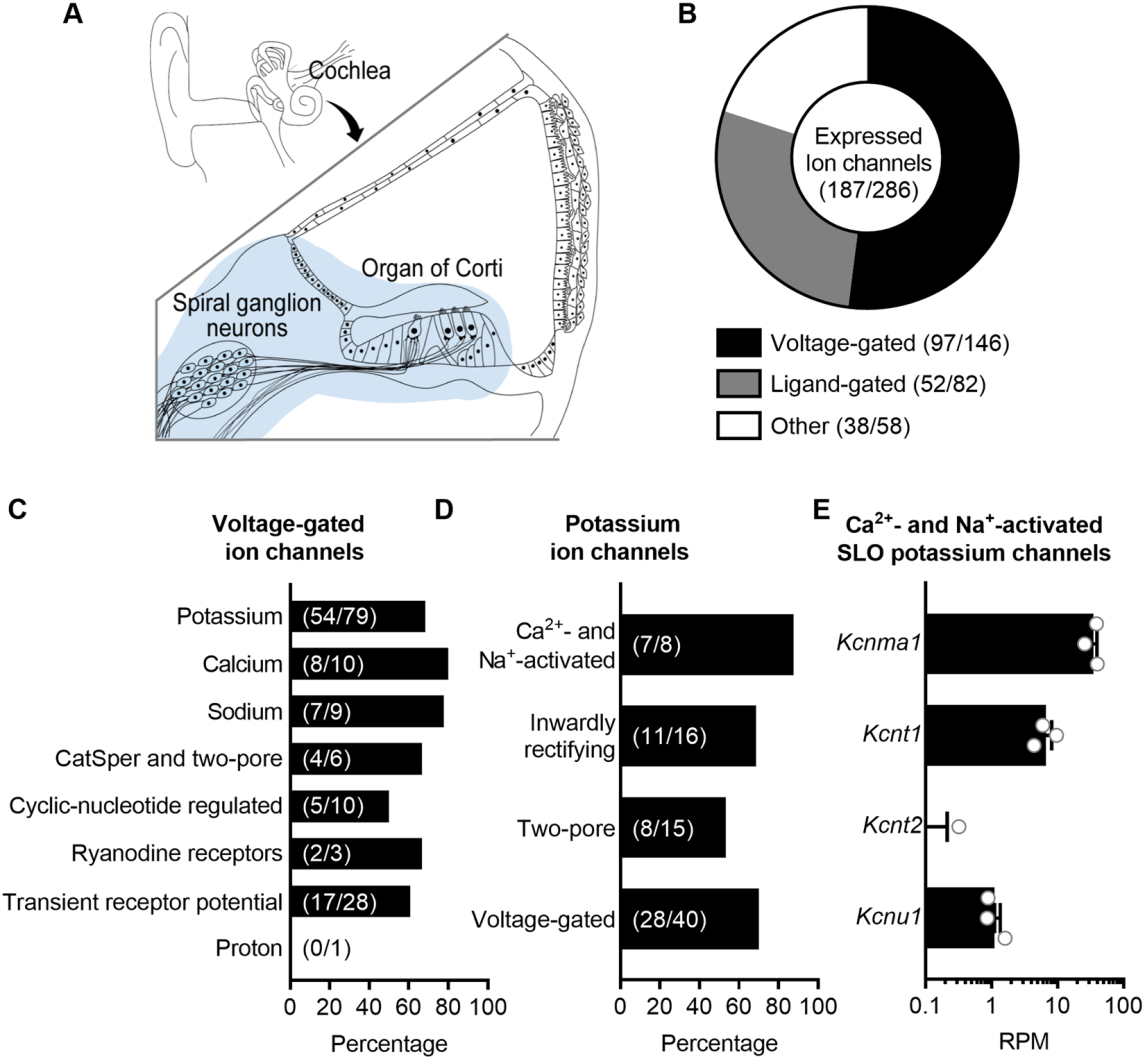
RNAseq identifies transcripts encoding the SLO channels K_Ca_1.1, K_Na_1.1, K_Na_1.2 but not K_Ca_5.1 in sensorineural structures of the mouse inner ear. (**A**) RNAseq was used to obtain whole transcriptomes from the sensorineural structures of the cochlea, including organs of Corti and spiral ganglion neurons (blue highlighted area), from post-hearing 6-week-old mice. (**B**) Following the classification of the IUPHAR/BPS Guide to Pharmacology, transcripts corresponding to a total of 65% of known ion channels (187/286 genes), subdivided into voltage-gated, ligand-gated and other ion channels, are expressed in the sensorineural structures. (**C**) The majority of transcripts encoding voltage-gated ion channels encode potassium channels, with transcripts corresponding to a total of 68% of known potassium channels (54/79 genes) expressed. (**D**) Of the potassium channels, 88% of the Ca^2+^- and Na^+^-activated potassium channels (7/8 genes) are expressed. (**E**) Three of the four members of the subset of Ca^2+^- and Na^+^-activated potassium channels encoding the SLO family of ion channels are expressed. For this group of ion channels, *Kcnma1*, which encodes K_Ca_1.1/SLO1/BK, is most abundantly expressed. *Kcnt1*, which encodes K_Na_1.1/SLO2.2/Slack, is expressed at intermediate levels. *Kcnt2*, which encodes K_Na_1.2/SLO2.1/Slick, is absent from two of the three replicates and expressed at <1 RPM in one replicate. *Kcnu1*, which encodes K_Ca_5.1/SLO3, is expressed at low levels. Data are plotted to show individual replicates (animals) and mean ± SEM. Values (mean ± SEM) are provided in the Results.

As part of a larger effort to identify the repertoire of ion channels that regulate encoding of auditory signals as part of the afferent signalling complex^3^, we used RNAseq to obtain whole transcriptomes from intact preparations of the organ of Corti and SGNs isolated from mice (Fig. 1). Following the classification of the IUPHAR/BPS Guide to Pharmacology, we mined these transcriptomes to determine expression of subsets of genes encoding for known voltage-gated ion channels (97/146 known genes expressed; Fig. 1A), potassium channels (54/79 genes; Fig. 1C), Ca^2+^- and Na^+^-activated potassium channels (7/8 genes; Fig. 1D) and, specifically, expression levels of genes encoding the SLO family of ion channels (Fig. 1E). For this group of K^+^ channels, *Kcnma1*, which encodes K_Ca_1.1/SLO1/BK, was most abundantly expressed (35.1 ± 4.7 RPM). *Kcnt1*, which encodes K_Na_1.1/SLO2.2/Slack, was expressed at intermediate levels (6.7 ± 1.5 RPM). *Kcnt2*, which encodes K_Na_1.2/SLO2.1/Slick, was absent from two of the three replicates and expressed at <1 RPM in one replicate. *Kcnu1*, which encodes K_Ca_5.1/SLO3, was expressed at ≈1 RPM (1.1 ± 0.2 RPM). To validate the utility of RNAseq to identify SLO transcripts in the sensorineural structures of the inner ear, we also examined the expression of K_Na_1-encoding transcripts in other tissues collected in parallel. RNAseq analyses revealed the following expression levels (in RPM): *Kcnt1:* 38 ± 1.7 (cerebellum), 0.18 ± 0.09 (heart) and 0 (liver); *Kcnt2*: 4.5 ± 0.82 (heart), 0 (cerebellum) and 0.060 ± 0.31 (liver). Thus, RNAseq analyses yields results consistent with qPCR detection of *Kcnt1* and *Kcnt2* in these tissues^19^. All values are expressed as mean ± SEM.

**Figure 2:**
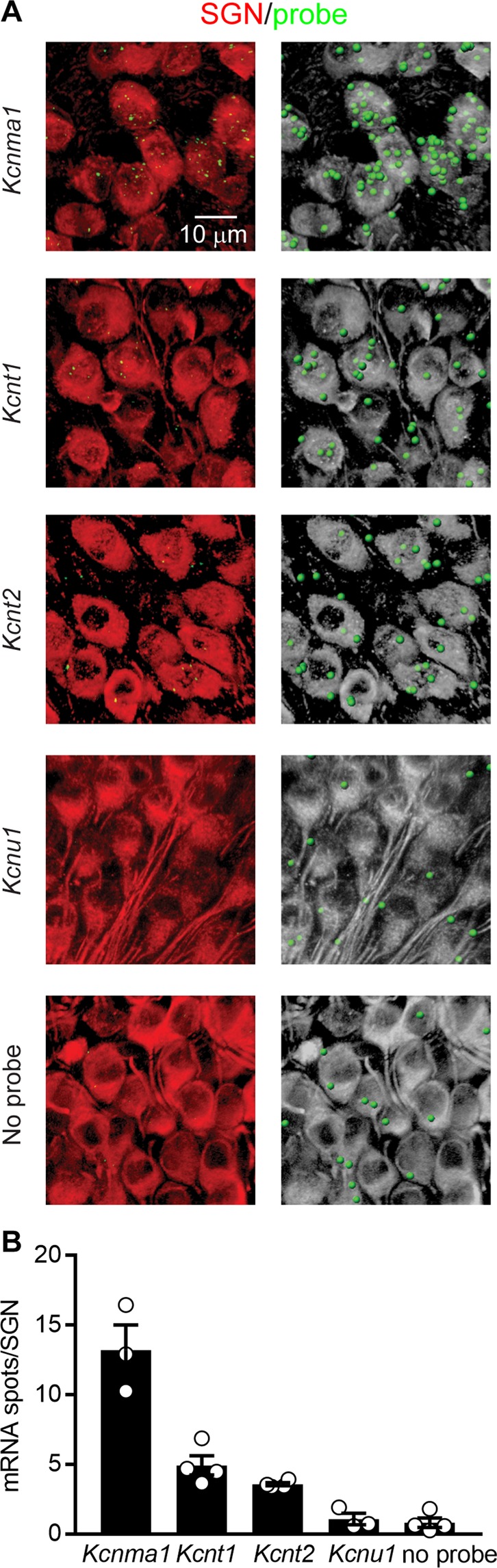
smFISH localizes transcripts encoding the SLO channels K_Ca_1.1, K_Na_1.1, K_Na_1.2 but not K_Ca_5.1 to the spiral ganglion neurons (SGNs). Expression of K_Na_1-encoding transcripts in the SGNs was examined using smFISH in excised preparations of the OC/SGN isolated from 6-week-old mice. (**A**) RNA molecules encoding for K_Ca_1.1 (*Kcnma1*), K_Na_1.1 (*Kcnt1*), K_Na_1.2 (*Kcnt2*), K_Ca_5.1 (*Kcnu1*) and no probe controls were detected as fluorescent puncta (green) in TUJ1-positive (red) SGN somas. For easier visualization of fluorescently labelled mRNA molecules, identical views are provided in which detected mRNA molecules are represented instead as spheres (green) and SGNs are shown in grey. (**B**) The mean number of RNA molecules detected per SGN was calculated as described in the Methods. *Kcnma1* was most abundantly expressed, *Kcnt1* and *Kcnt2* were expressed at intermediate values, and K*cnu1* and no probe controls showed little to no expression. Data are plotted to show individual replicates (animals) and mean ± SEM. Values (mean ± SEM) are provided in the Results.

We suspected that K_Na_1-encoding transcripts in the sensorineural structures were specifically expressed by the SGNs for two reasons. First, K_Na_1 channels have been observed in other primary sensory neurons^16,19,21–24^. Second, activation of K_Na_1 channels requires rises in intracellular Na^+^ mediated by activation of TTX-sensitive, voltage-gated Na^+^ channels and/or ionotropic AMPA-type glutamate receptors^12^. SGNs express TTX-sensitive (persistent and resurgent) voltage-gated Na^+^ channels^4,25^ as well as AMPA-type glutamate receptors^26,27^. In contrast, mature hair cells do not express voltage-gated Na^+^ currents^28,29^ or glutamate receptors. To examine cell-specific expression of K_Na_1 channels in the organ of Corti and SGNs, we utilized a variety of commercially available antibodies against these channels. Unfortunately, none of these antibodies yielded consistent or reliable results (data not shown). Therefore, as an alternative strategy, we used single molecule fluorescent *in situ* hybridization (smFISH) to localize expression of K_Na_1-encoding transcripts in cochlear sections (Fig. 2). Transcripts encoding for K_Ca_1.1, K_Na_1.1 and K_Na_1.2 *(Kcnma1*, *Kcnt1* and *Kcnt2*) but not K_Ca_5.1 *(Kcnu1)* were detected (as green particles) in the TUJ1-labeled (red) SGN somas (Fig. 2A). To quantify relative transcript abundance, the total number of RNA molecules detected per SGN was calculated across independent replicates (Fig. 2B). *Kcnma1* (encoding K_Ca_1.1) was richly expressed, *Kcnt1* and *Kcnt2* (encoding K_Na_1.1 and K_Na_1.2) were expressed at intermediate values, and *Kcnu1* (encoding K_Ca_5.1) and no probe controls showed little to no expression (*Kcnma1*: 13 ± 1.8 mRNA/SGN, n = 3 replicates; *Kcnt1*: 4.9 ± 0.69 mRNA/SGN, n = 4 replicates; *Kcnt2*: 3.6 ± 0.12 mRNA/SGN, n = 4 replicates; *Kcnu1*: 1.1 ± 0.42 mRNA/SGN, n = 3 replicates; no probe: 0.82 ± 0.34 mRNA/SGN, n = 4 replicates). All values are expressed as mean ± SEM. Together, smFISH and RNAseq analyses suggest that of the two K_Na_1-encoding transcripts, K_Na_1.1-encoding transcripts are more abundantly expressed in the sensorineural structures and specifically SGNs. Differences in the relative expressions of these two transcripts between the two techniques most likely arises from dilution of transcript expression in the intact preparation used for RNAseq. Importantly, observation of K_Na_1-encoding transcripts in the sensorineural structures and specifically SGNs of the cochlea motivated *in vivo* examination of the contribution of K_Na_1 channels to peripheral auditory function.

### K_Na_1 DKO mice have normal ABR thresholds but reduced wave I amplitudes

**Figure 3:**
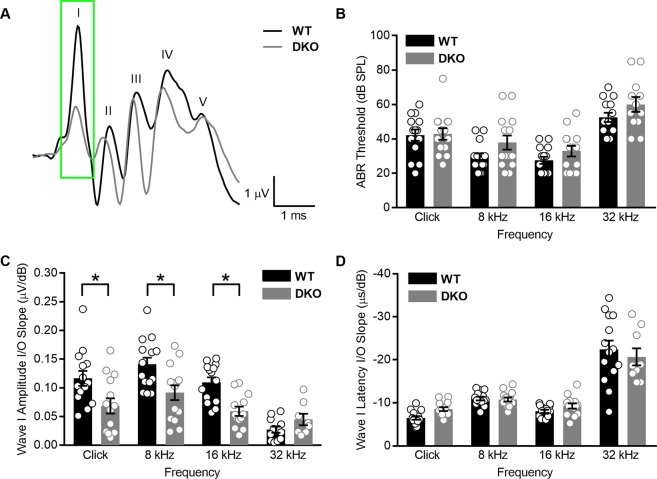
K_Na_1 DKO mice have normal ABR absolute thresholds but reduced wave I responses. Auditory brainstem responses (ABR) were measured in 6-week-old WT and DKO mice. (**A**) Raw traces of ABRs to suprathreshold sound intensities (90 dB SPL) are shown in response to sound clicks for both genotypes. (**B**) Mean absolute ABR thresholds in response to click and tone pips at 8, 16 and 32 kHz were not statistically significantly different between WT and DKO mice. (**C**) Mean wave I amplitude I/O linear regression slopes were significantly reduced in DKO mice compared to WT mice in response to click and tone pips at 8 and 16 kHz. (**D**) Wave I latency I/O linear regression slopes were not significantly different between WT and DKO mice. Data are plotted to show individual replicates (animals) and mean ± SEM. Values (mean ± SEM) are provided in Table 1. Statistical analyses are provided in the Results.

To examine the contribution of K_Na_1 channels to peripheral auditory function, we recorded auditory brainstem responses (ABRs) from WT and K_Na_1.1/1.2 DKO mice. ABRs provide a non-invasive electrophysiological measure of auditory function. ABR wave I results from action potentials from the auditory nerve and are diagnostic for sensorineural hearing loss. We specifically investigated K_Na_1.1/1.2 DKO mice to avoid potential compensation of one channel type for the other. Example raw traces of suprathreshold ABRs (measured at 90 dB SPL) are shown in response to sound clicks for both genotypes (Fig. 3A). ABR analyses revealed that absolute ABR thresholds, that is the sound intensity where wave I is consistently discernible above noise, were not statistically significantly different between WT and DKO mice when comparing between sound stimuli (Fig. 3B; p-values = 0.9997 for clicks, 0.2123 for 8 kHz, 0.6547 for 16 kHz and 0.3725 for 32 kHz, ordinary one-way ANOVA with Sidek’s correction for multiple comparisons). In addition to absolute thresholds, we also examined wave I amplitudes and latencies as a function of sound intensity. For both WT and DKO mice, ABR waveform amplitudes increased and latencies decreased as stimulus intensity increased. To compare changes between genotypes and sound stimuli, we calculated I/O linear regression slopes for wave I amplitudes (Fig. 3C) and latencies (Fig. 3D) as a function of stimulus intensity. Wave I amplitude I/O linear regression slopes were significantly reduced in response to click and tone pips at 8 and 16 kHz in DKO compared to WT mice (p-values = 0.0078 for clicks, 0.0061 for 8 kHz, 0.0061 for 16 kHz and 0.8047 for 32 kHz, ordinary one-way ANOVA with Sidek’s correction for multiple comparisons). Although mean values for wave I latency I/O linear regression slopes were reduced in response to click and tone pips at 16 kHz in DKO compared to WT mice, these differences were not statistically significant (p-values = 0.4501 for clicks, 0.9998 for 8 kHz, 0.8392 for 16 kHz and 0.7198 for 32 kHz, one-way ANOVA with Sidek’s correction for multiple comparisons). Mean values ± SEMs are provided in Table 1. These results indicate that function of the auditory nerve is altered in K_Na_1 DKO mice. In fact, DKO mice show a form of hearing loss termed “hidden hearing loss”, which is characterized by normal thresholds but reduced suprathreshold wave I responses. Hidden hearing loss has been documented in both animal models and humans and is thought to precede overt hearing loss, which is detectable (or “unhidden”) as elevations in absolute auditory thresholds^30^. Thus, we further characterized K_Na_1.1/1.2 DKO mice to gain insight into the molecular and cellular contributions of KNa1 channels to normal auditory function and the mechanisms underlying this form of hidden hearing loss.

**Table 1:** ABR values from 6-week-old WT and K_Na_1 DKO mice.

Measure	Thresholds (dB peSPL)		Wave I Amplitude I/O slopes (µV/dB)		Wave I Latency I/O slopes (−µs/dB)	
Genotype	WT	DKO	WT	DKO	WT	DKO
N	14	12–14	12–14	8–14	14	9–14
Click	42 ± 3	43 ± 3	0.117 ± 0.013	0.068 ± 0.013	6.5 ± 0.4	8.5 ± 0.4
8	29 ± 2	38 ± 4	0.141 ± 0.012	0.092 ± 0.013	11 ± 0.4	10.8 ± 0.5
16	28 ± 2	33 ± 3	0.109 ± 0.009	0.059 ± 0.008	8.0 ± 0.4	9.3 ± 0.6
32	53 ± 3	60 ± 4	0.027 ± 0.005	0.045 ± 0.010	22.4 ± 2.1	21 ± 2.0

### Cochlear morphology, spiral ganglion cell density, and architecture of the afferent synapses are normal in K_Na_1 DKO mice

**Figure 4:**
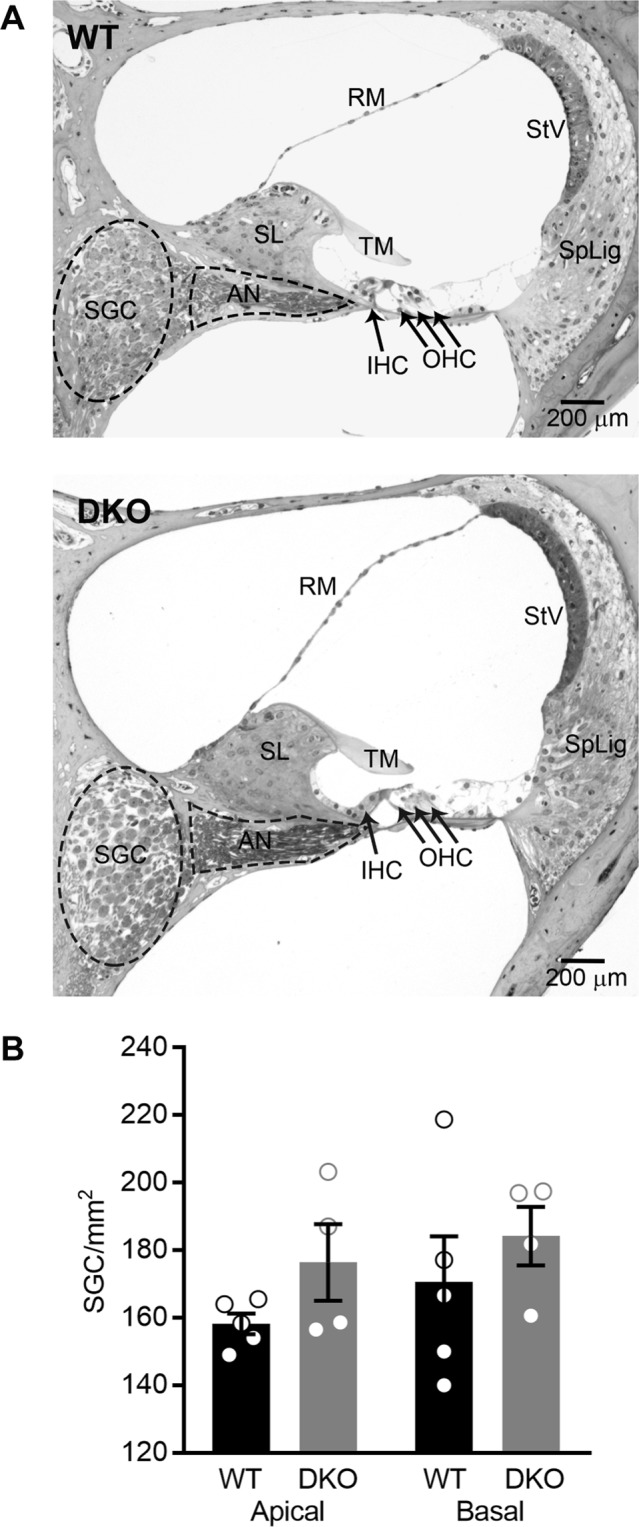
Cochlear morphology and spiral ganglion cell (SGC) density are normal in K_Na_1 DKO mice. Cochlear morphology and SGC density were examined in mid-modiolar serial sections through the cochlea isolated from 6-week-old WT and DKO mice. (**A**) No differences between genotypes were seen in structures of the inner ear, including hair cells, SGCs, stria vascularis, spiral ligament and all supporting structures of the cochlear duct. (**B**) Spiral ganglion cell-density was not significantly different between WT (black) and DKO (grey) mice in either cochlear apical or basal turns. Data are plotted to show individual replicates (animals) and mean ± SEM. Values (mean ± SEM) and statistical analyses are provided in the Results.

In both animal models and humans, overt hearing loss is associated with loss of sensorineural structures and “hidden” hearing loss is particularly associated with loss of synapses between the sensory inner hair cells (IHCs) and SGNs^30–33^. Therefore, we assessed both the morphology of the sensorineural structures and the integrity of the synapses between the IHCs and SGNs in DKO compared to WT mice. Spiral ganglion cell (SGC) density and overall cochlear morphology was examined in mid-modiolar serial sections in cochleae isolated from (6-week-old) WT and DKO mice (Fig. 4). No abnormalities were seen in the cells and tissues of the cochlea, including the inner and outer hair cells, SGCs, stria vascularis, spiral ligament and all supporting structures of the cochlear duct (Fig. 4A). Importantly, both the packing density of the SGCs as well as the density of auditory nerve fibers were visually comparable between WT and DKO mice. When SGC density was quantified, no statistically significant differences were observed between WT and DKO mice in either cochlear apical (WT: 158 ± 3 SGC/mm^2^, n = 5; DKO: 176 ± 11 SGC/mm^2^, n = 4, p value = 0.7827, one-way ANOVA with Sidek’s correction for multiple comparisons) or basal segments (WT: 171 ± 14 SGC/mm^2^, n = 5; DKO: 184 ± 9 SGC/mm^2^, n = 4, p value = 0.9278, one-way ANOVA with Sidek’s correction for multiple comparisons).

**Figure 5:**
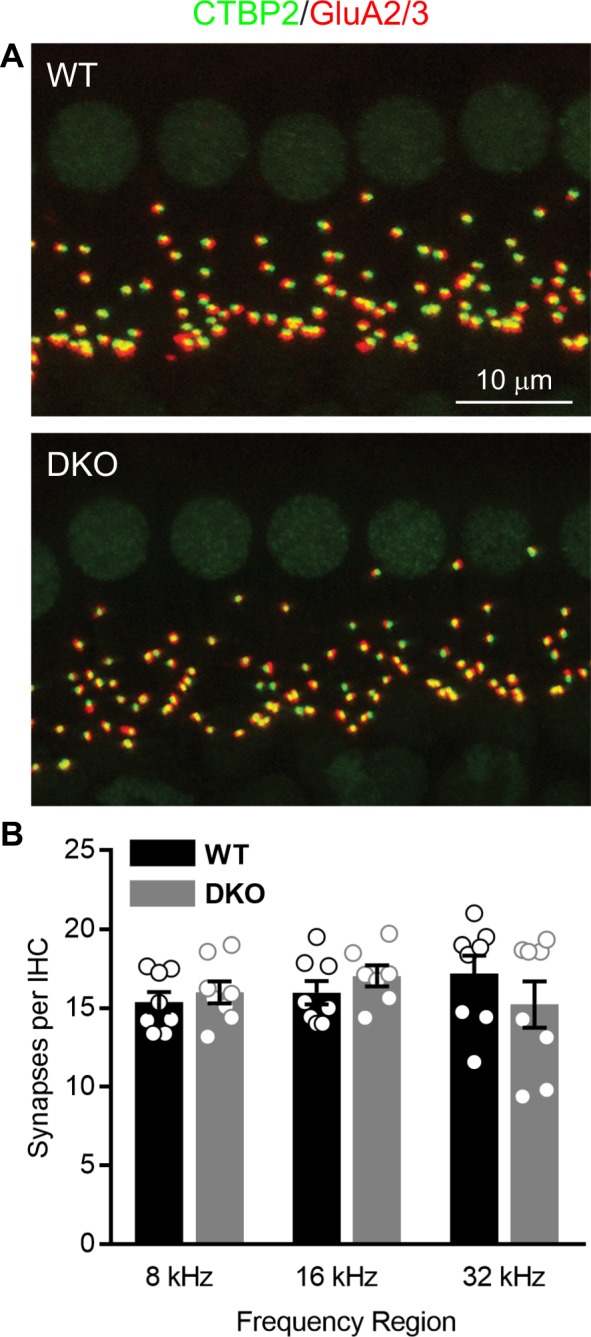
Cochlear afferent synapse counts are normal in K_Na_1 DKO mice. Synapses between the spiral ganglion neurons (SGNs) and inner hair cells (IHCs) were quantified at three tonotopic locations (8, 16 and 32 kHz) in organs of Corti isolated from 6-week-old WT and DKO mice. (**A**) There were no obvious differences between WT and DKO mice in the organization of afferent synapses, identified as paired CtBP2 (green) and GluR2/3-(red) immunopuncta. Images are presented as Z-projections through a stack of confocal micrographs from the 16 kHz region. (**B**) Quantification of the mean number of synapses per IHC indicated no statistically significant differences between WT (black) and DKO (grey) mice at any of the tonotopic regions. Data are plotted to show individual replicates (animals) and mean ± SEM. Values (mean ± SEM) are provided in Table 2. Statistical analyses are provided in the Results.

Because the SGNs can show delayed loss after much earlier loss of synaptic contacts to the IHCs^30^, we also examined the synaptic connections between the IHCs and SGNs at three tonotopic locations (8, 16 and 32 kHz) in intact preparations of the organ of Corti and SGNs isolated from (6-week-old) WT and DKO mice (Fig. 5). There were no observable qualitative differences between WT and DKO mice in the organization of afferent synapses, identified as paired CtBP2 (green) and GluR2/3 (red) immunopuncta (Fig. 5A). Quantification of the mean number of synapses per IHC indicated no statistically significant differences between WT and DKO mice when comparing between tonotopic regions (Fig. 5B; p-values = 0.9543 for 8 kHz, 0.8315 for 16 kHz and 0.3834 for 32 kHz, ordinary one-way ANOVA with Sidek’s correction for multiple comparisons). Mean values ± SEMs are provided in Table 2. Thus, despite differences in the ABR wave I responses between WT and DKO mice, which mimic “hidden” hearing loss in the DKO mice, there were no indications of morphological alterations or synaptopathy in the cochleae of DKO mice.

**Table 2:** Synapses per IHC from 6-week-old WT and K_Na_1 DKO mice.

	8 kHz		16 kHz		32 kHz	
	WT (n = 8)	DKO (n = 8)	WT (n = 8)	DKO (n = 7)	WT (n = 8)	DKO (n = 8)
Mean ± SEM	15.4 ± 0.7	16.0 ± 0.7	16.0 ± 0.7	17.0 ± 0.7	17.2 ± 1.1	15.2 ± 1.5
N (IHCs)	59	81	77	57	56	83
N (immunopuncta)	893	1,295	1,212	964	957	1,217

**Figure 6:**
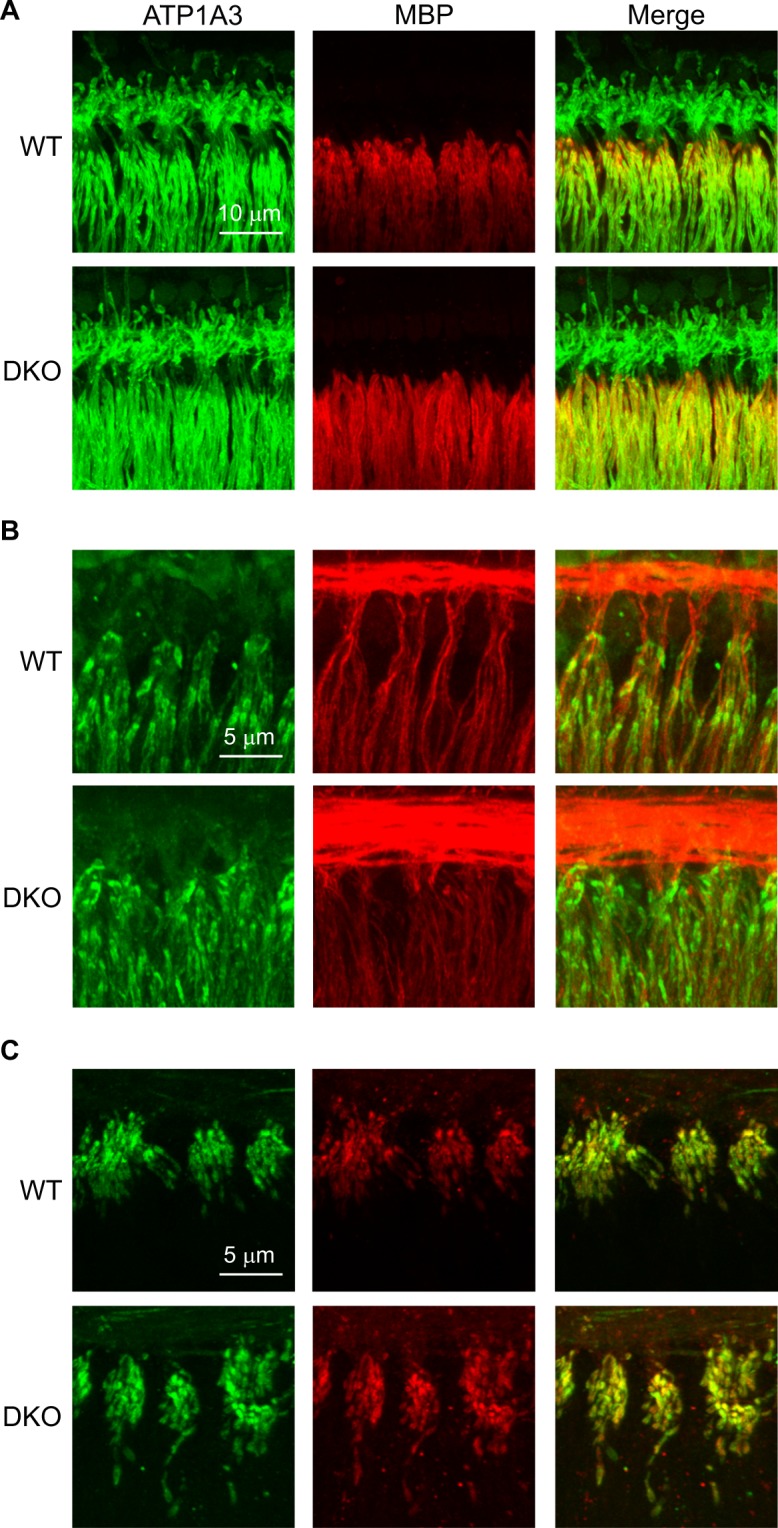
Molecular and cellular architecture appears normal in SGNs from K_Na_1 DKO mice. The expression and distribution of various proteins shaping SGN excitability were examined using immunofluorescence in the isolated preparation of the organ of Corti and SGNs from (6-week-old) WT and DKO mice. (**A**) The expression of the Na^+^, K^+^-ATPase α3 (ATP1A3, green) and patterns of myelination were similar in both WT and DKO mice. (**B**) The expression and distribution of the low voltage-activated K_V_1.1 (green) was similar in both WT and DKO mice. Tubulin J (TuJ, red) marks the SGN afferent dendrites and is provided for reference. (**C**) The expression and distribution of the high voltage-activated K_V_3.1 (green) and the voltage-gated Na_V_1.6 (red) were similar in both WT and DKO mice. All images are presented as Z-projections through a stack of confocal micrographs from the 16 kHz region. Expression patterns of ATP1A3, MBP, K_V_1.1, K_V_3.3 and Na_V_1.6 were similarly expressed at other regions as well as in the somata of the SGNs from WT and DKO mice.

These findings suggested that the phenotype of hidden hearing loss in the DKO mice may result from changes other than synaptopathy in the SGNs. Therefore, we examined the expression and distribution of various proteins positioned to shape SGN excitability using immunofluorescence in the isolated preparation of the organ of Corti and SGNs from (6-week-old) WT and DKO mice. We particularly examined placement of these proteins at the SGN afferent dendrites, where synapses are made between the SGNs and IHCs and where spike generation occurs. The precise alignment of proteins here is expected to underlie SGN excitability and perhaps also firing synchrony^34^. We examined expression of the Na^+^, K^+^-ATPase α3 (ATP1A3, green, Fig. 6A), a transporter expressed in the SGNs^35^ and known to regulate neuronal excitability^36^, the patterns of myelination indicated by distribution of myelin basic protein^37^ (MBP, red, Fig. 6A), and the expression of various ion channels known to support SGN firing, including the low voltage-activated K_V_1.1^38–40^ (green, Fig. 6B), the high voltage-activated K_V_3.1^41,42^ (green, Fig. 6C) and the voltage-gated Na_V_1.6^43,44^ (red, Fig. 6C). We found no visible evidence of altered ATP1A3 expression or patterns of myelination in the DKO compared to WT mice. Moreover, we found no visible evidence of altered expression or localization of K_V_1.1, K_V_3.3 or Na_V_1.6 compared to previous topographical characterization^34^. In both WT and DKO mice, K_V_1.1 was localized to heminodes and nodes and K_V_3.1 and Na_V_1.6 were colocalized at heminodes and nodes. These data were collected from the mid-cochlear (16 kHz) region. Although not shown, expression patterns of ATP1A3, MBP, K_V_1.1, K_V_3.3 and Na_V_1.6 were similarly expressed at other regions as well as in the somata of the SGNs from WT and DKO mice. These findings indicate that loss of K_Na_1 channels does not alter the molecular and cellular architecture of proteins critical for shaping SGN responses *in vivo*. These findings, in turn, motivated our investigation of possible alterations in the physiology of SGNs lacking K_Na_1 channels.

### Spiral ganglion neurons isolated from K_Na_1 DKO mice do not have Na^+^-sensitive outward K^+^ currents and display altered action potential waveforms

**Figure 7:**
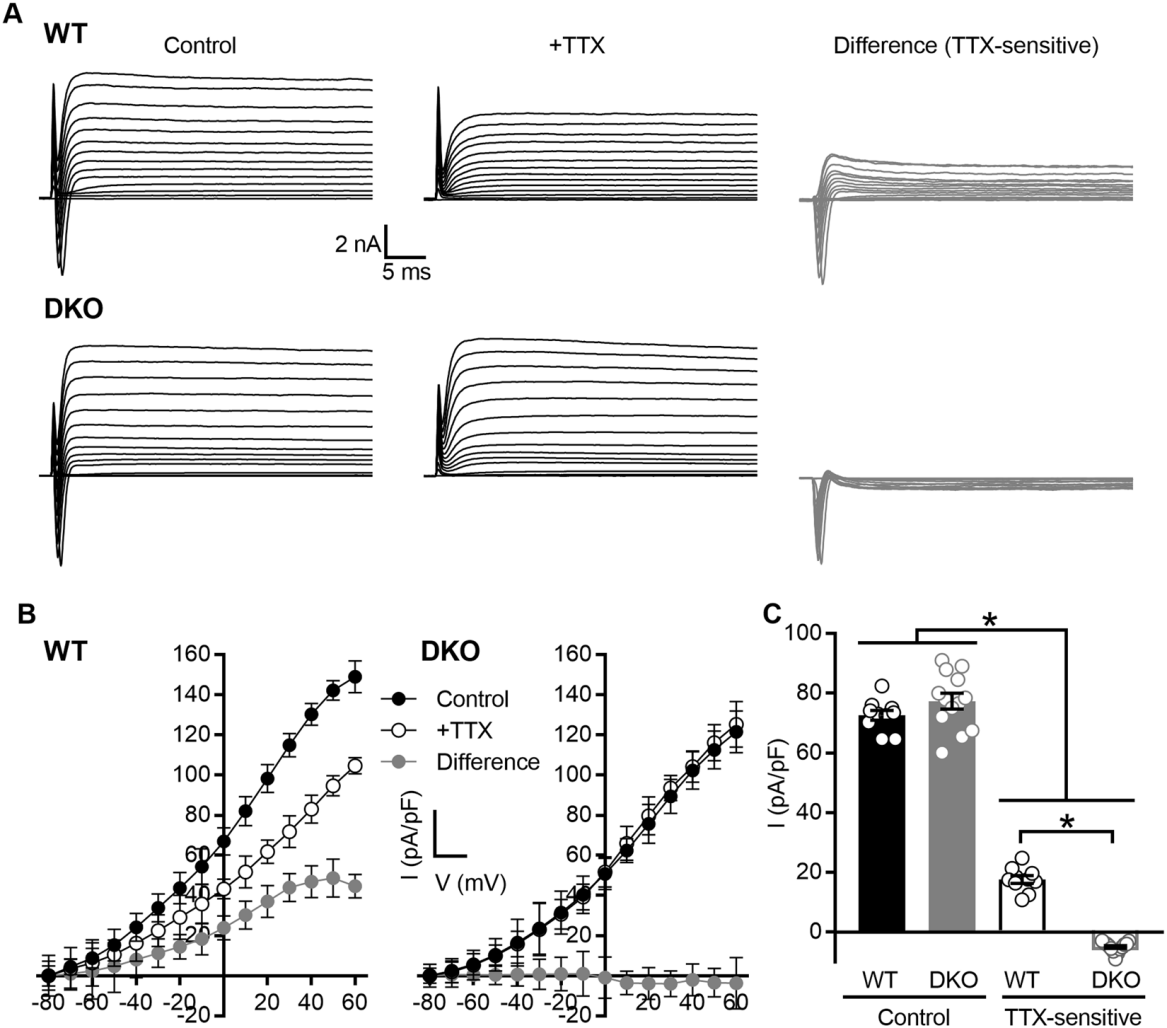
Na^+^-sensitive outward K^+^ currents are absent in spiral ganglion neurons (SGNs) isolated from K_Na_1 DKO mice. Whole cell patch clamp recordings were performed on SGNs isolated from the basal one-third of the cochlea from 6-week-old WT and DKO mice. (**A**) Whole-cell currents revealed a transient inward current and also outward currents in SGNs isolated from both WT and DKO mice in response to varying depolarization from a holding potential of −80 mV to 60 mV in 10-mV increments (control). Bath application of 1 µM TTX completely blocked voltage-sensitive inward currents in SGNs isolated from both WT and DKO mice (+TTX). The difference current generated by subtraction of currents recorded in the presence of TTX from those recorded before TTX application (control) revealed the TTX-sensitive outward currents in SGNs isolated from WT and DKO mice (difference, TTX-sensitive). (**B**) To compare findings across SGNs, the current density-voltage relationship was generated using the steady state K^+^ current amplitude for both WT and DKO mice. (**C**) The mean TTX-sensitive outward K^+^ current density calculated at 0 mV was significantly greater in SGNs from WT compared to DKO mice. Data are plotted to show individual replicates (animals) and mean ± SEM. Values (mean ± SEM) and statistical analyses are provided in the Results.

To investigate directly the contribution of K_Na_1 channels to SGN responses, we performed whole cell patch clamp recordings on SGNs isolated from (6-week-old) WT and DKO mice. The use of genetic models circumvents the lack of pharmacological tools to block K_Na_1 channels^12^ and/or methodological approaches that require comparison of recordings from separate cells with different internal Na^+^ concentrations^17,19,45^. In the voltage-clamp configuration, whole-cell currents revealed a transient inward current, mediated by voltage-dependent inward (Na^+^) current, as well as outward currents in SGNs isolated from both WT (Fig. 7A, control) and DKO (Fig. 7B, control) mice in response to varying depolarization from a holding potential of −80 mV to 60 mV in 10-mV increments. Bath application of 1 µM TTX completely blocked voltage-sensitive inward currents in SGNs isolated from both WT (Fig. 7A, +TTX) and DKO (Fig. 7B, +TTX) mice. The difference-current generated by subtraction of currents recorded in the presence of TTX from those recorded before TTX application (control) revealed the TTX-sensitive currents in SGNs isolated from WT (Fig. 7A, difference) and DKO (Fig. 7B, difference). These difference currents consisted of a fast inward current and a slow outward current. The TTX-sensitive inward current arises from activation of voltage-dependent Na^+^ channels, whereas the outward component is inferred to arise from the Na^+^-activated K^+^ channels. From a total of 31 basal SGNs isolated from WT mice, 16 expressed sizable TTX-sensitive K^+^ currents, ranging from 10 to 32% of the total outward current. The remaining 15 SGNs expressed substantially less TTX-sensitive K^+^ current (approximately 2–5% of the total outward current). In contrast, SGNs isolated from DKO mice were always devoid of TTX-sensitive K^+^ currents. To quantify findings across basal SGNs, the current density-voltage relationship was generated using the steady state K^+^ current amplitude for both WT mice (Fig. 7C, n = 10 cells from which both voltage and current clamp data were collected) and DKO mice (Fig. 7D, n = 13 cells). The total outward K^+^ current density at 0 mV (Fig. 7E) was 72.6 ± 1.7 pA/pF in SGNs from WT mice (n = 10) and not significantly different from the total outward K^+^ current density of 77.3 ± 2.6 pA/pF in SGNs from DKO mice (n = 13; p value = 0.3348, ordinary one-way ANOVA with Sidek’s correction for multiple comparisons). In contrast, when present, the mean TTX-sensitive outward K^+^ current density at 0 mV was 17.6 2 ± 1.3 pA/pF in SGNs from WT mice (n = 10) and significantly greater than the mean TTX-sensitive outward K^+^ current density at 0 mV in SGNs from DKO mice (5.0 ± 0.5 pA/pF, n = 13; p value < 0.0001, ordinary one-way ANOVA with Sidek’s correction for multiple comparisons).

Similar findings were also observed when recording from SGNs isolated from the apical one-third of the cochlea from (6-week-old) WT and DKO mice. For 23 apical SGNs, 14 SGNs had a mean total outward current density at 0 mV of 73.0 ± 10.0 pA/pF. The mean TTX-sensitive K^+^ current at 0 mV was calculated to be 14.7 ± 6.0 pA/pF (or approximately 20% of the total outward current). The remaining 9 neurons expressed substantially less TTX-sensitive K^+^ current (approximately 2–5% of the total outward current). Again, SGNs isolated from DKO mice showed no measurable TTX-sensitive K^+^ currents. Similar findings were also observed when extracellular Na^+^ was replaced by either Li^+^, which permeates voltage-gated Na^+^ channels but would not be expected to activate K_Na_1 channels fully^46^ or by NMG^+^, a bulkier monovalent cation that is less likely to permeate Na^+^ channels and activate Na^+^-dependent K^+^ currents. Both Li^+^ and NMG^+^-sensitive K^+^ current densities calculated at 0 mV for SGNs isolated from WT mice (Li^+^: 8.7 ± 0.6 pA/pF, n = 10; NMG: 4.5 ± 0.5 pA/pF, n = 10) were greater than the equivalent currents calculated for SGNs isolated from DKO mice (Li^+^: −2.7 ± 0.4 pA/pF, n = 13; NMG: −1.5 ± 0.2 pA/pF, n = 13). Similar subtractive methods and Na^+^ substitution have been used previously to identify K_Na_1 currents^45–47^.

Together these data indicate the presence of Na^+^-activated K^+^ currents in approximately half of both apical and basal SGNs isolated from WT mice. The absence of this current in DKO mice is consistent with the current being carried by K_Na_1 channels. Furthermore, the reduction of this current in SGNs from WT mice when voltage-gated Na^+^ channels are blocked by TTX or when Na^+^ is replaced by either Li^+^ or NMG, strongly suggest that the K_Na_1 channels in SGNs are activated, at least in part, by Na^+^ influx most likely via the TTX-sensitive voltage-dependent Na^+^ channels.

**Figure 8:**
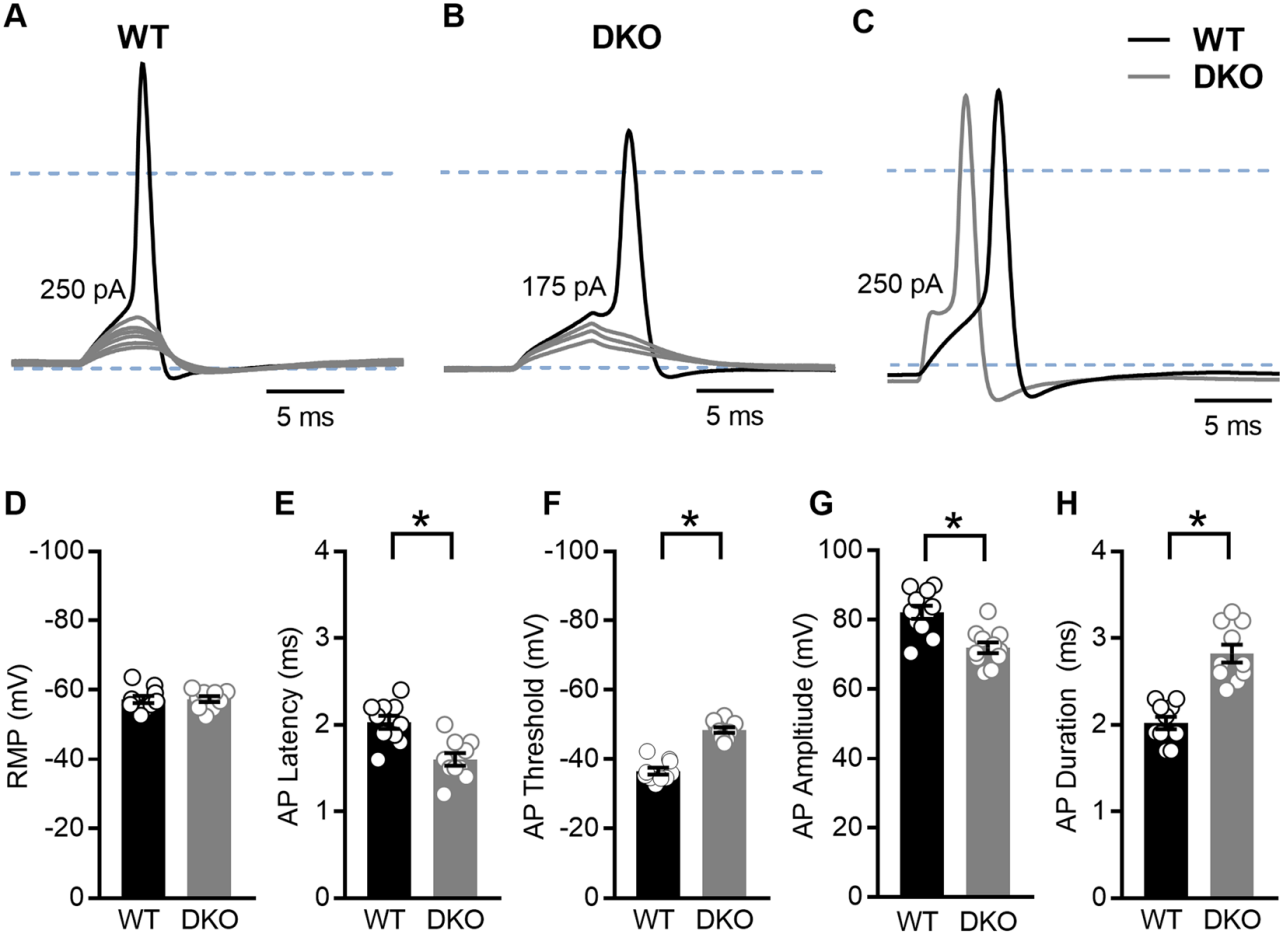
Properties of evoked action potentials (APs) are altered in spiral ganglion neurons (SGNs) isolated from K_Na_1 DKO mice. To examine the effects of K_Na_1 on membrane properties in SGNs, current-clamp recordings were performed on SGNs isolated from the basal one-third of the cochlea from 6-week-old WT and DKO mice. (**A**) SGNs isolated from WT mice required greater current injection to evoke action potentials (APs) compared to SGNs isolated from DKO mice. (No DC current was injected to set the resting potential for different cells to the same value.) (**B**) Comparatively, APs evoked in SGNs from WT mice (black traces) were generally slower to initiate and larger in amplitude compared to those evoked in SGNs from DKO mice (grey traces). Dotted blue lines indicate −60 mV and 0 mV. (**C**–**G)** Across SGNs, the resting membrane potential (RMP) was not significantly different between WT and DKO mice (**C**). In contrast, significant differences in the AP latency, (**D**), membrane potential threshold for AP generation (**E**), AP amplitude (**F**) and AP duration (**G**) were observed between SGNs from WT and DKO mice. Data are plotted to show individual replicates (animals) and mean ± SEM. Values (mean ± SEM) from basal and apical SGNs are provided in Tables 3 and 4. For these data, significant differences between genotypes were determined using unpaired two-tailed t test.

In other neurons, K_Na_1 currents contribute to setting the resting membrane potential, shaping the action potential waveform and altering repetitive firing^12,13^. To examine the effects of K_Na_1 channels on membrane properties in SGNs, we performed current-clamp recordings on the majority of SGNs also examined by voltage-clamp recordings. In this way we could compare SGNs from DKO mice with SGNs that express K_Na_1 currents from WT mice. In general, while resting membrane potentials appeared similar in SGNs from both genotypes, SGNs isolated from WT mice (Fig. 8A) required increased current injection to evoke an action potential compared to SGNs isolated from DKO mice (Fig. 8B). Comparatively, action potentials evoked in SGNs from WT mice were generally slower to initiate and larger in amplitude compared to those evoked in SGNs from DKO mice (Fig. 8C). Quantifying across cells, the resting membrane potentials of SGNs isolated from basal cochlear turns of WT and DKO mice were not significantly different (Fig. 8D, p value = 0.9333, unpaired, two-tailed t test). Nevertheless, active membrane properties did significantly differ between SGNs from DKO compared to WT mice (Fig. 8E–H). In general, SGNs isolated from DKO compared to WT mice were more excitable, showing significantly reduced action potential latencies (Fig. 8E, p value = 0.0007, unpaired, two-tailed t test) and thresholds (Fig. 8F, p value < 0.0001, unpaired, two-tailed t test). Additionally, action potential amplitudes were significantly decreased in SGNs from DKO compared to WT mice (Fig. 8G, p value = 0.0004, unpaired, two-tailed t test) whereas action potential durations (measured as the width at half-maximal spike amplitude) were significantly increased in SGNs from DKO compared to WT mice and durations (Fig. 8H, p value < 0.0001, unpaired, two-tailed t test). Similar trends were observed in SGNs isolated from the apical one-third of the cochlea, with the exception that apical SGNs from DKO mice also showed more negative resting membrane potentials (RMPs) compared to SGNs from WT mice. Values are provided in Tables 3 and 4. These data suggest, that across frequencies, the presence of K_Na_1 channels in SGNs normally serves to delay action potential generation, increase the threshold of action potential generation, and increase action potential amplitude.

**Table 3:** Membrane properties of SGNs isolated from the basal one-third of the cochlea from 6-week-old WT and K_Na_1 DKO mice.

RMP (mV) (n = 10)		Latency (ms) (n = 10)*		AP Threshold (mV) (n = 10)*		AP Amplitude (mV) (n = 11)*		AP Duration (ms) (n = 10)*	
WT	DKO	WT	DKO	WT	DKO	WT	DKO	WT	DKO
−57 ± 1.0	−57 ± 0.83	2.0 ± 0.08	1.6 ± 0.07	−37 ± 0.95	−48 ± 0.80	82 ± 1.9	72 ± 1.5	2.0 ± 0.07	2.8 ± 0.10

**Table 4:** Membrane properties of SGNs isolated from the apical one-third of the cochlea from 6-week-old WT and K_Na_1 DKO mice.

RMP (mV)* (n = 10)		Latency (ms) (n = 10)*		AP Threshold (mV) (n = 10)*		AP Amplitude (mV) (n = 11)*		AP Duration (ms) (n = 10)*	
WT	DKO	WT	DKO	WT	DKO	WT	DKO	WT	DKO
−59 ± 0.95	−64 ± 1.3	2.3 ± 0.08	1.9 ± 0.07	−38 ± 1.2	−49 ± 0.78	79 ± 1.4	71 ± 1.3	2.1 ± 0.08	2.4 ± 0.09

## Discussion

To accelerate the discovery of K^+^ channels that regulate encoding of auditory signals, we used RNA sequencing to obtain transcriptomes from the intact sensorineural structures, including the organ of Corti and SGNs, isolated from adult (6-week-old) mice. Transcriptomic analyses revealed a vast repertoire of K^+^ channels positioned to regulate encoding of auditory signals. In fact, of the tissues investigated by transcriptomic analyses, K^+^ channel diversity was greatest in the organs of Corti and SGNs (with 68% or 54/80 genes known to encode K^+^ channels expressed) compared to cerebellum (65% or 49/80 genes), heart (59% or 47/80 genes) and liver (35% or 28/80 genes). In prioritizing K^+^ channels for further investigation, we were especially interested in the expression of the SLO family of ion channels and particularly the sodium-activated K^+^ channels, K_Na_1.1 and K_Na_1.2. These channels are unique in their regulation by intracellular [Na^+^], and recent work has identified their central role in regulating neuronal function, development and plasticity^12^.

In this work, expression analysis indeed identified K_Na_1-encoding transcripts in the sensorineural structures of the organ of Corti and SGNs (by RNAseq) and specifically localized transcript expression to the SGNs (by smFISH). Multiple independent observations corroborate SGN-specific expression of K_Na_1-encoding transcripts and K_Na_1 channels. First, K_Na_1-encoding transcripts (*Kcnt1* and *Kcnt2*) have been identified in earlier microarray expression analyses of FACS-sorted mature (P15) SGNs^48^ and absence of both *Kcnt1* and *Kcnt2* in FACS-sorted mature (P16) hair cells^49^. Although a previous microarray study examining isolated mature (P25–30) hair cells detected low expression of *Kcnt1* and *Kcnt2* in both the inner and outer hair cells^50^, a subsequent more sensitive RNAseq study found no expression of either gene in the mature hair cells^51^. Second, K_Na_1 currents and K_Na_1-encoding transcripts have been previously detected in rat vestibular ganglion neurons using a combination of electrophysiology and reverse-transcription PCR^47^. Immunofluorescence indicated the presence of K_Na_1 in the somata and dendrites of the vestibular ganglion neurons and the absence of K_Na_1 in the vestibular hair cells. Third, the expression of K_Na_1-encoding transcripts and currents specifically in the SGNs and not hair cells is consistent with the neuronal distribution of K_Na_1 channels in the central nervous system^12^ and lack of sources for Na^+^ influx in the mature hair cells^28,29,52^. Finally, previous microarray datasets indicate increasing expression of the K_Na_1-encoding transcripts *Kcnt1* and *Kcnt2* in FACS-sorted SGNs from E12 to P15^48^. Thus, K_Na_1 currents could have been missed in earlier investigations, which more routinely examine immature SGNs^53^.

To investigate the contribution of K_Na_1 channels to peripheral auditory function, we took advantage of K_Na_1 DKO mice. We observed normal absolute thresholds but reduced suprathreshold wave I amplitudes in K_Na_1 DKO compared to WT mice. This phenotype mimics a recently identified form of hearing loss coined hidden hearing loss that has been attributed to loss of the synapses between the sensory inner hair cells and SGNs^30^. However, our anatomical characterization indicates that DKO mice have normal numbers of SGNs and normal numbers of synapses between the SGNs and IHCs. Moreover, the molecular and cellular architecture of proteins critical for shaping SGN excitability also appears normal in SGNs from DKO mice. These findings suggest that physiological rather than anatomical properties of the SGNs underly the phenotype of hidden hearing loss in these mice. For these reasons, we examined excitability of isolated SGNs using patch clamp electrophysiology.

Electrophysiological characterization of the SGNs revealed three consistent findings in SGNs isolated from both apical and basal cochlear turns. First, we observed expression of K_Na_1 currents in some but not all SGNs isolated from WT mice. Second, we found reduced thresholds for action potential firing in SGNs isolated from K_Na_1 DKO mice compared to SGNs expressing K_Na_1 currents from WT mice. Third, action potential amplitudes were reduced in SGNs isolated from DKO mice compared to SGNs expressing K_Na_1 currents from WT mice. Together, these three findings suggest that K_Na_1 currents are normally expressed in a subset of SGNs, where they serve to increase action potential thresholds and amplitudes of those SGNs.

Although extrapolation of results obtained from SGNs *in vitro* to auditory nerve responses *in vivo* must be done cautiously, changes in the response properties of SGNs isolated from K_Na_1 DKO mice may contribute to the phenotype of hidden hearing loss observed in these mice. Specifically, differences in threshold sensitivity among SGNs have been documented in a variety of mammals^54–57^. These variations in threshold sensitivity enable encoding of sound intensities over a large dynamic range: SGNs with lower thresholds contribute to auditory nerve (wave I) responses to lower sound intensities (near threshold), whereas SGNs with higher thresholds contribute to responses to higher (suprathreshold) sound intensities^31^. Thus, the reduced growth of auditory nerve (wave I) responses to suprathreshold sound intensities observed in K_Na_1 DKO mice may result from the diminished contribution of a subpopulation of SGNs with higher action potential thresholds. Consistent with this reasoning, noise exposure that causes hidden hearing loss, the phenotype mirrored by K_Na_1 DKO mice, appears to result specifically in loss of auditory nerve fibers with high thresholds^58,59^. Moreover, the reduced growth of auditory nerve (wave I) responses to suprathreshold sound intensities observed in K_Na_1 DKO mice may also result from the reduced action potential amplitudes of a subpopulation of SGNs. Finally, we found normal expression and distribution of ion channels essential for action potential generation in the SGNs from K_Na_1 DKO mice. Moreover, no other K^+^ channels could replicate the Na^+^-activation of K_Na_1 channels. Nevertheless, we cannot rule out the possibility that there may be quantitative differences in expression and/or functional changes in the properties of these or other ion channels that lead to the altered responses we observed *in vivo* and/or *in vitro* in response to genetic deletion of K_Na_1.

This work also raises questions for future consideration about the regulation of K_Na_1 channels in both SGNs and neurons more broadly. First, future studies should take advantage of SGNs to identify the sources of intracellular Na^+^ ions required for activation of K_Na_1 currents. During the voltage steps used in our experiments (<100 ms), the TTX-sensitive outward K^+^ currents in SGNs showed little degradation. Na^+^ influx through transient Na^+^ channels can produce sustained activation of K_Na_1 currents if the two channels are coupled intimately and local Na^+^ concentration remains elevated. Alternatively or additionally, K_Na_1 current in SGNs may be activated by Na^+^ influx via persistent Na^+^ channels that have been identified previously in mouse SGNs^25^. In either case, our findings suggest close coupling of the voltage-gated Na^+^ channels and K_Na_1 channels, which would allow Na^+^ influx during the upstroke of the action potential to activate K_Na_1 currents and modify properties of action potential threshold and latency in ways we observed in this report. Indeed, activation of K_Na_1 currents during action potential initiation has been proposed by others^19^. Examination of the interaction of Na^+^ influx via voltage-gated Na^+^ channels and K_Na_1 channel activation may also provide insight into the mechanisms underlying the reduced action potential amplitudes we unexpectedly observed in SGNs isolated from K_Na_1 DKO mice.

Second, future experiments should utilize SGNs to examine mechanisms that regulate K_Na_1 channel expression and activity. In this study, electrophysiological examination revealed K_Na_1 currents in a subset of SGNs. In contrast, smFISH analyses indicates expression of K_Na_1-encoding transcripts in all SGNs. Similarly, recent work using single-cell RNA sequencing to define three subtypes of (type I) SGNs, reported expression of *Kcnt1* in all three SGN subtypes^60^. These findings suggest that expression of K_Na_1 channels in subpopulations of SGNs is regulated post-transcriptionally. Indeed, a variety of intracellular signalling cascades and neuromodulators are known to modulate K_Na_1 channel activity in other cell types, including neurons of the dorsal root ganglion^16,24,61–65^. These mechanisms may give rise to the differences in K_Na_1 channel activity between subpopulations of SGNs within a specific cochlear region, between apical and basal SGNs, and between SGNs and other neurons.

Third, our work highlights the need for further investigation of K_Na_1 in SGNs to deepen our understanding of the molecular mechanisms that contribute to heterogeneous responses of the auditory neurons and correlated susceptibilities to excytotoxic damage. More specifically, our observation of a phenotype that mimics hidden hearing loss without synapse loss in the K_Na_1 DKO mice suggests that the anatomical synaptopathy underlying hidden hearing loss may, in some cases, be preceded by physiological changes in SGN excitability. If so, identification of the molecular cascades by which the absence of K_Na_1 channels exert these changes in SGN excitability and, in turn, their possible link to synapse loss and hearing loss, will inform the development of treatments that intervene in the earliest stages in the progression of hearing loss.

In conclusion, our results show that K_Na_1 channels are part of a much larger repertoire of K^+^ channels positioned to regulate the primary auditory neurons. This work provides an efficient strategy to identify, prioritize and characterize the contributions of these K^+^ channels to function of the peripheral auditory system both *in vitro* and *in vivo*.

## Methods

### Animals

All experimental protocols were approved and carried out in accordance with the relevant guidelines and regulations in place at the University Medical Center Groningen (UMCG) and the University of Nevada Reno. K_Na_1 double knockout (DKO) mice were bred onto a C57BL/6 background for 12 generations^19^. Because utilization of wildtype (WT) littermates were not feasible, age and gender matched C57BL/6 mice were obtained from either the C57BL/6 stock maintained at the UMCG Central Animal Facility or The Jackson Laboratory. No comparative differences between WT C57BL/6 from these two sources were observed. Nevertheless, whole genome scanning was performed to confirm strain identity and assess genetic quality between stocks. The C57BL/6 J sub-strain was confirmed via single nucleotide polymorphism (SNP)-based genome scanning (performed by Jackson Laboratories). 100% of the 150 SNP markers evenly spaced over the 19 autosomes and the X chromosome were identical in the C57BL/6 J colony maintained at the UMCG Central Animal Facility compared to the sub-strain maintained by Jackson Laboratories (data not shown).

### RNA isolation and sequence analysis

#### Micro-dissection of cochlear tissue

Mice were anaesthetized with isoflurane before being sacrificed by decapitation. All mice were male and sacrificed at the same time of day to avoid hormonal and circadian variations in transcript expression between replicates. Cochleae were isolated from the temporal bones in ice-cold phosphate buffered solution (PBS). Cochlear tissue was micro-dissected, with the organ of Corti with SGNs saved separately from the lateral wall tissue (the stria vascularis with the spiral ligament). Micro-dissection was performed without decalcification or other pre-treatment (including harsh mechanical or chemical lysis), and care was taken to remove the overlying bone and associated vasculature as much as possible, including the red blood cell niche at the apex of the cochlea. Micro-dissected tissues were immediately transferred to ice-cold TRIzol reagent and processed for RNA isolation. For comparison, cerebellum, heart and liver were simultaneously collected and identically prepared.

#### RNA isolation

Micro-dissected tissues were homogenized in TRIzol reagent using a rotor-stator homogenizer. RNA extraction was performed using the ARCTURUS PicoPure RNA Isolation Kit with the addition of a DNase treatment. RNA quality and quantity were verified with a ThermoFisher Nanodrop. Samples with highest RNA quantity were checked for RNA quality by capillary electrophoresis using a Perkin Elmer LabChip GX. Samples with distinct 18S and 28S peaks were chosen for RNA sequencing.

#### RNA sequencing

RNA sequencing (RNAseq) and quality control (QC) were performed by the Genome Analysis Facility (GAF) at the UMCG. Illumina TrueSeq RNA sample preparation kits were used to generate sequence libraries using the Perkin Elmer Sciclone NGS Liquid Handler. cDNA fragment libraries were sequenced on an Illumina HiSeq2500 (single reads 1 × 50 bp) in pools of multiple samples. A total of 3 independent replicates (from 3 mice) were analyzed. The *Mus musculus* GRCm38 Ensembl Release 82 reference genome was used to align trimmed fastQ files with hisat. Sorting of aligned reads was performed using SAMtools. Gene level quantification was performed by HTSeq and Ensembl version 82 was used as gene annotation database. FastQC was used for QC measurements of raw sequencing data. Picard-tools calculated QC metrics for aligned reads. Sequence counts were standardized against total number of high quality reads for each sample. Because only one fragment was sequenced per transcript, length normalization was not necessary. For each gene, the mean values were generated from three replicate standardized values. Transcripts were considered present only if 2 of the 3 reads were greater than 0 RPM.

### Single molecule fluorescence *in situ* hybridization (smFISH) with RNAscope

### Preparation of cochlear sections

Mice were anesthetized with an intraperitoneal injection of ketamine (100 mg/kg) and xylazine (10 mg/kg) and then transcardially perfused with diethyl pyrocarbonate (DEPC)-treated phosphate-buffered saline (PBS) and 4% paraformaldehyde (PFA) in 0.1 M phosphate buffer. The cochleae were harvested and immersed in a 4% paraformaldehyde (PFA) solution (DEPC-treated) overnight on a shaker at 4 °C. The cochleae were washed with PBS and decalcified in 0.35 M ethylenediaminetetraacetic acid (EDTA) for 5 days on a shaker at 4 °C and washed with PBS. Samples were cryoprotected by sequential immersion in 10%, 20%, and 30% sucrose solution at 4 °C for 1 h, 2 h, and overnight, respectively. Samples were transferred into optimal cutting temperature (OCT) compound for a minimum of 1 hr at 4 °C and then snap frozen, using a dry ice-ethanol mixture. Samples were cryo-sectioned to a thickness of 12 μm, placed onto Superfrost slides and stored at −80 °C until further use.

### Probe hybridization and subsequent immunofluorescent staining

Probe hybridization closely followed the manufacturer’s instructions (Advanced Cell Diagnostics). Sections were immersed in pre-chilled 4% PFA for 15 min at 4 °C. They were then dehydrated at room temperature (RT) in 50%, 70% and 100% ethanol (2X) for 5 min each and allowed to dry for 1–2 min. Fixation and dehydration was followed by protease digestion, using Protease 4 for 30 min at RT. Sections were then incubated at 40 °C with the following solutions: 1) target probe in hybridization buffer A for 3 hours; 2) preamplifier in hybridization buffer B for 30 minutes; 3) amplifier in hybridization buffer B at 40 °C for 15 minutes; and 4) label probe in hybridization buffer C for 15 minutes. After each hybridization step, slides were washed with wash buffer three times at RT. For fluorescent detection, the label probe was conjugated to Alexa Fluor 488. Probes for K^+^ channels and a blank negative control were obtained from Advanced Cell Diagnostics. Sequences of the target probes (for the specified K^+^ channels), preamplifier, amplifier, and label probe are proprietary. Detailed information about the probe sequences can be obtained by signing a nondisclosure agreement provided by the manufacturer.

For subsequent immunofluorescent staining, slides were treated with 10% blocking solution for 10 min at RT, incubated with anti-Tubulin β3 (TUJ1, BioLegend, 1:300 dilution), overnight at 4 °C, washed with PBS three times for 5 min each, incubated with the appropriate Alexa Fluor secondary antibody (ThermoFisher) diluted 1:500 for 2 hours at RT, and again washed with PBS three times for 5 min each. Incubation in Hoechst 33342 solution for 15 s at RT was performed to label cell nuclei. Slides were then mounted in Fluoromount-G and sealed under a coverslip.

#### Imaging and image analysis

Confocal micrographs were obtained as described below. Individually fluorescently labelled mRNA transcripts appeared as puncta. To quantify the number of mRNA transcripts per SGN, individually fluorescently labelled mRNAs within a given field of view (FOV) were detected using the spots function in Imaris 6.4 software (Bitplane). mRNA counts were normalized to the number of TUJ1-labeled SGNs marked manually in the same FOV.

### Measurement of auditory brainstem responses

Mice were anesthetized with an intraperitoneal injection of 75 mg/kg ketamine and 1 mg/kg dexmedetomidine and placed in an acoustic chamber. ABRs were recorded in response to both click and pure tone pips (8, 16, 32 kHz) stimuli produced with an open field speaker as described previously^66^. Responses were averaged over 512 recordings. P1, N1, P2 and N2 were detected manually blind to genotype and frequency and used to calculate wave I and II amplitudes and latencies. Input-output (I/O) function slopes of the amplitude and latency growth function curves (that is, amplitude and latency as a function of stimulus intensity) were calculated as described previously^67–70^ over stimulus intensities ranging from 40–90 dB SPL. I/O function slopes were only calculated when distinct positive and negative peaks could be unambiguously identified.

### Histological assessment of the cochlear morphology

Mice were anesthetized with an intraperitoneal injection of 300 μg/g Avertin, (2,2,2-tribromethanol) and transcardially perfused with PBS followed by a fixative solution, containing 4% PFA and 2% glutaraldehyde in 0.1 M cacodylate buffer (pH 7.4). The cochlea was isolated, perilymphatically perfused with and then immersed in the fixative overnight at room temperature. The cochleae were post-fixed with 1% osmium tetroxide, decalcified with 120 mM EDTA at 23 °C for 48 h, and then dehydrated and embedded in an epoxy resin. Semi-thick (1 µm) sections of the cochleae were cut in the mid-modiolar plane and stained with toluidine blue for examination by light microscopy. Images (20×) were captured using a Nikon Eclipse 80i microscope. Spiral ganglion cells from the lower basal and apical segments were quantified from four to five images spaced 100 µm apart. Final figures were assembled using Adobe PhotoShop and Illustrator software (Adobe Systems).

### Immunofluorescence, confocal microscopy and image analysis of isolated auditory sensory epithelia

Mice were anaesthetized with isoflurane before being sacrificed by decapitation. Cochleae were isolated from the temporal bones in ice-cold phosphate buffered solution (PBS) and then fixed for 1 to 3 hours in a fixative solution containing 4% PFA. Auditory sensory epithelia were isolated and immunostained as described previously^35^. The primary antibodies used in this study included anti-C-terminal-binding protein 2 (CtBP2, mouse IgG1, BD Biosciences 612044), anti-glutamate A-receptor 2/3, (GluR2/3, rabbit polyclonal, Millipore AB1506), anti Na,K-ATPase α3 (ATP1A3, mouse IgG1, ThermoFisher MA3-915), anti-myelin basic protein (MBP, mouse IgG2b, Covance SMI-99), anti K_V_1.1 (rabbit polyclonal, Alomone Labs APC-009), anti-tubulin J (TUJ, mouse IgG2a, Covance TUJ1), anti K_V_3.1 (rabbit polyclonal, Alomone Labs APC-014), and anti-Na_V_1.6 (mouse IgG1, NeuroMab 75-026) and were used diluted 1:300. The appropriate Alexa Fluor secondary antibodies (ThermoFisher) were used diluted 1:500. To determine frequency regions in isolated organs of Corti, low magnification micrographs of organs of Corti were obtained using a Leica DM4000B fluorescent microscope. If necessary, the Stitching plugin in ImageJ was used to create a single montage image. Tonotopic maps were then overlaid on the image using a specially developed plugin in Image J (https://www.masseyeandear.org/research/otolaryngology/investigators/laboratories/eaton-peabody-laboratories/epl-histology-resources/imagej-plugin-for-cochlear-frequency-mapping-in-whole-mounts) and the previously determined place-frequency map of the mouse cochlea^71^. High magnification confocal micrographs were collected using a Leica SP8 confocal microscope with a 63× oil immersion lens under the control of the LAS X software. Z-stacks of the entire inner hair cell (IHC) synaptic pole from the 8, 16 and 32 kHz region were collected at a scan speed of 200 Hz and zoom of 1. The step size (optical section thickness) was determined by stepping at half the distance of the theoretical z-axis resolution (the Nyquist sampling frequency). Images were acquired in a 1024 × 1024 raster (x = y = 184.52 µm × 184.52 µm) at sub-saturating laser intensities for each channel. Images are presented as z-projections through the collected optical stack. All quantitative image analysis was performed on the raw image stacks, without deconvolution, filtering, or gamma correction. The number of synaptic elements per IHC were determined from 3D reconstructions generated using Imaris 6.4 software (Bitplane) as described previously^72^. Final figures were assembled using Adobe PhotoShop and Illustrator software (Adobe Systems).

### Patch clamp electrophysiology of isolated spiral ganglion neurons

#### Isolation of spiral ganglion neurons (SGNs)

SGNs were isolated from male and female WT and K_Na_1 DKO mice as described in detail previously^53,73–75^. Mice were anaesthetized and the temporal bones were removed in a solution containing Minimum Essential Medium with Hank’s salt (Invitrogen), 0.2 g/L kynurenic acid, 10 mM MgCl_2_, 2% fetal bovine serum (FBS; v/v), and 6 g/L glucose. The SGN tissue was dissected and split into three equal segments: apical, middle and basal segments across the modiolar axis. Apical and basal thirds were used to obtain viable neuronal yield for experiments. Additionally, tissue was pooled from three mice into each SGN culture. The apical and basal tissues were digested separately in an enzyme mixture containing collagenase type I (1 mg/mL) and DNase (1 mg/mL) at 37 °C for 20 min. After a series of gentle trituration and centrifugation in 0.45 M sucrose, the cell pellets were reconstituted in 900 mL culture media (Neurobasal-A, supplemented with 2% B27 (v/v), 0.5 mM L-glutamine, 100 U/mL penicillin) and filtered through a 40-µm cell strainer for cell culture. SGNs were cultured for 24 to 48 h to allow detachment of Schwann cells from neuronal membrane surfaces. All electrophysiological experiments were performed at RT (21–22 °C).

#### Voltage- and current-clamp experiments

Whole-cell current and voltage-clamp recordings of action potentials and ionic currents, respectively, were performed at room temperature as described earlier^76,77^ using an Axopatch 200B amplifier. For current clamp recordings, the fast current clamp mode was used. Electrodes (2–3 MΩ) were pulled from borosilicate glass pipettes, and the tips were fire-polished. Extracellular/bath solution contained (in millimolar) 130 NaCl (or 130 LiCl or 130N-methyl-D-glucamine (NMG)Cl), 5 KCl, 1 MgCl_2_, 2 CaCl_2_, 10 D-glucose, and 10 Hepes, pH 7.3. The normal pipette/internal solution contained (in millimolar) 112 KCl, 2.5 EGTA, 1 MgCl_2_, 0.01 CaCl_2_, 5 ATP-K_2_, and 10 HEPES, pH 7.3. Considering [EGTA] and both [ATP] and [Mg^2+^], free [Ca^2+^] in the internal solution was determined using the MaxChelator program (http://maxchelator.stanford.edu/CaMgATPEGTA-TS.htm) and estimated to be <1 nM. Current traces were generated with depolarizing voltage steps from a holding potential of −80 mV and stepped to varying positive potentials (ΔV = 5–15 mV). At this holding potential, at least 60 to 70% of the total voltage-activated Na^+^ channel current is expected to be activated. The seal resistance was typically 5–10 GΩ. Currents were measured with capacitance and series resistance compensation (>90%), filtered at 2 kHz using an 8-pole Bessel filter and sampled at 5 kHz. In all cases, liquid junction potentials were measured and corrected as described previously^78^. The capacitive transients were used to estimate the cell capacitance and, in turn, provide an indirect measure of cell size. Cell capacitance was approximately 21.8 ± 5.0 pF (n = 37). Whole-cell inward and outward current amplitudes at varying test potentials were measured at the peak and steady-state levels using a peak detection routine; the current magnitude was divided by the cell capacitance (pF) to determine the current density-voltage relationship. The stock solutions of tetrodotoxin (TTX) were made in ddH_2_O and stored at −20 °C.

## Data Availability

The datasets generated during and/or analysed during the current study are available from the corresponding author on reasonable request.
